# Thermal Analysis of a Reactive Variable Viscosity TiO_2_-PAO Nanolubricant in a Microchannel Poiseuille Flow

**DOI:** 10.3390/mi14061164

**Published:** 2023-05-31

**Authors:** Oluwole Daniel Makinde, Anuoluwa Esther Makinde

**Affiliations:** 1 Faculty of Military Science, Stellenbosch University, Private Bag X2, Saldanha 7395, South Africa; 2 Faculty of Engineering, Stellenbosch University, Private Bag X1, Matieland 7602, South Africa; anu.makinde7@gmail.com

**Keywords:** microchannel, nanolubricant hydrodynamics, variable viscosity, thermal analysis

## Abstract

This paper examines the flow structure and heat transfer characteristics of a reactive variable viscosity polyalphaolefin (PAO)-based nanolubricant containing titanium dioxide (TiO_2_) nanoparticles in a microchannel. The nonlinear model equations are obtained and numerically solved via the shooting method with Runge–Kutta–Fehlberg integration scheme. Pertinent results depicting the effects of emerging thermophysical parameters on the reactive lubricant velocity, temperature, skin friction, Nusselt number and thermal stability criteria are presented graphically and discussed. It is found that the Nusselt number and thermal stability of the flow process improve with exothermic chemical kinetics, Biot number, and nanoparticles volume fraction but lessen with a rise in viscous dissipation and activation energy.

## 1. Introduction

Polyalphaolefins (PAO) are the major synthetic lubricants that are commonly used in various industrial and automotive applications. They are typically less volatile and are designed to provide superior lubrication performance over a wider operating temperature range [1]. The advent of nanofluids ensuing from nanotechnology has also yielded a major improvement in industrial heat transfer processes, cooling technologies, and tribological properties in many applications such as machines and engines [2]. Nanolubrication, therefore, can be defined as the art and science necessary to control adhesion, friction, and wear of surfaces coming into contact at the micro/nano-scale. The primary purpose of dispersing nanoparticles in lubricants is to enhance engine performance and energy efficiency, increase resilience to extreme pressure, lessen wear and improve heat transfer from the friction zone [3]. In addition, fuel and lubricant consumption are cut, and harmful emissions are reduced. Recently, several researchers and scientists [4,5,6,7] have conducted both experimental and theoretical studies on nanolubricants due to their tremendous applications. Borda et al. [8] experimentally examined the tribological behaviour of nanolubricants containing copper as nanoparticles. The heat dissipation effects of oil-based nanolubricants in a refrigeration system were theoretically and experimentally studied by Choi et al. [9]. Nayak et al. [10] numerically analysed the hydromagnetic boundary layer convection of ZnO-SAE50 nanolubricant past an inclined rotating disk with heat transfer characteristics. Pico et al. [11] experimentally studied the heat transfer behaviour of TiO_2_-R600a nano-refrigerant in a domestic refrigerator. Gamaoun et al. [12] reported the impact of ZnO-SAE50 nanolubricant on the heat transfer behaviour of a convection–conduction fin. 

Meanwhile, it is well known that the most sensitive lubricant’s property to temperature is viscosity [13]. As the temperature gets very hot, lubricant viscosity sufficiently drops and may undergo chemical changes that can drastically reduce its effectiveness and life expectancy. Conversely, as the temperature gets very cold, lubricant viscosity considerably increases, thus, losing its ability to lubricate effectively. Therefore, it is critical to always take the operating temperature of the equipment with lubricants into account [14]. In addition, most lubricants used in engineering and industrial processes, including polyalphaolefins (PAO) lubricants, contain hydrocarbons and are reactive in the presence of oxygen and other reactive chemicals [15,16]. Equipment failure, friction between parts, and excessive exothermic kinetics can serve as the ignition point during operation, leading to thermal runaway [17]. Therefore, adequate safety precautions are necessary in handling such nanolubricants in order to prevent loss of life and properties. 

A review of the literature shows that no study Is reported yet on the thermal analysis of a reactive variable viscosity TiO_2_-PAO nanolubricant in a microchannel. Titanium dioxide (TiO_2_) is a promising lubricant additive capable to enhance engine efficiency [18]. This investigation is aimed at filling this gap. The steady flow and heat transfer characteristics of a reactive variable viscosity TiO_2_-PAO nanolubricant in a microchannel with exothermic chemical kinetics and convective conditions at the upper wall is investigated. In microchannel nanolubricant hydrodynamics, Poiseuille flow scenarios may occur. For instance, in a fluid film bearing nanolubrication opposing surfaces are completely separated by a nanolubricant film under an applied pressure gradient, leading to Poiseuille flow parabolic velocity profiles within the microchannel. The applied load is carried by pressure generated within the nanolubricant, and the frictional resistance to motion arises entirely from the shearing of the nanolubricant [19,20,21]. Under the fluid film lubrication regime, both friction and wear are minimised. In the following sections, the model problem is formulated, analysed, and numerically tackled. Pertinent results are displayed graphically and discussed.

## 2. Model Problem

**Figure 1:**
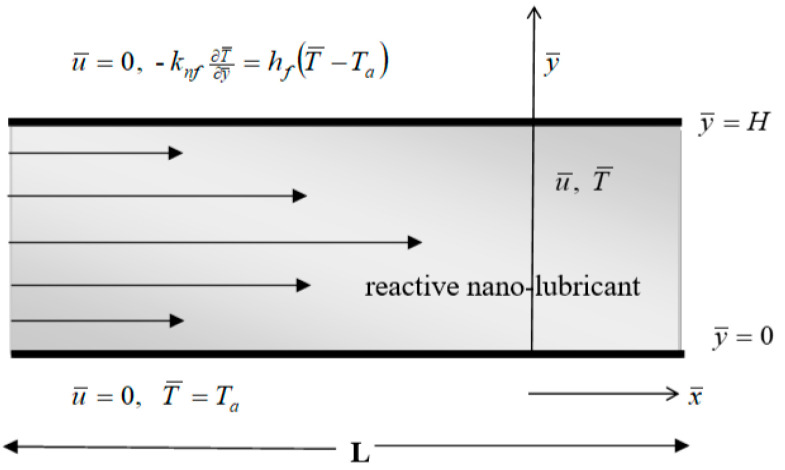
Schematic diagram of the problem.

Consider the steady flow of a variable viscosity reactive polyalphaolefins (PAO)-based nanolubricant containing titanium dioxide (TiO_2_) nanoparticles in a fairly long microchannel of width *H* and length *L*. The microchannel lower wall is maintained at ambient temperature *T_a_* while the upper wall convectively exchanges heat with the ambient surrounding. During the flow process, it is assumed that the exothermic Arrhenius kinetics may take place in the nanolubricant and the heat transfer coefficient is *h_f_*. The microchannel configuration is aligned with the *x*-axis where the *y*-axis is normal to it, as shown in Figure 1 below.

Under these conditions, the continuity, momentum, and energy equations governing the problem in dimensionless form may be written as [5,14,19,20,21]

(1) $$\frac{\partial u}{\partial x}+\frac{\partial v}{\partial y}=0,$$

(2) ε2ReA1(u∂u∂x+v∂u∂y)=−∂p∂x+2ε2A2∂∂x(e1β(1+βT)∂u∂x)+A2∂∂y[e1β(1+βT)(∂u∂y+ε2∂v∂x)],

(3) ε4ReA1(u∂v∂x+v∂v∂y)=−∂p∂y+2ε2A2∂∂y(e1β(1+βT)∂v∂y)+ε2A2∂∂x[e1β(1+βT)(∂u∂y+ε2∂v∂x)],

(4) $$\varepsilon^{2}PeA_{3}\left(u\frac{\partial T}{\partial x}+v\frac{\partial T}{\partial y}\right)=A_{4}\left(\varepsilon^{2}\frac{\partial^{2}T}{\partial x^{2}}+\frac{\partial^{2}T}{\partial y^{2}}\right)+A_{2}\Phi e^{\frac{1}{\beta (1+\beta T)}}+\lambda e^{-\frac{1}{\beta (1+\beta T)}},$$

where

(5) $$\Phi =Ec\mathrm{Pr}\left[2\varepsilon^{2}{\left(\frac{\partial u}{\partial x}\right)}^{2}+2\varepsilon^{2}{\left(\frac{\partial v}{\partial y}\right)}^{2}+{\left(\frac{\partial u}{\partial y}+\varepsilon^{2}\frac{\partial v}{\partial x}\right)}^{2}\right].$$

The appropriate boundary conditions in dimensionless form are given as follows:

(6) u=0, T=0, aty=0,

(7) $$u=0,\;A_{4}\frac{\partial T}{\partial y}=-BiT,\;\mathrm{at}y=1.$$

The following dimensionless quantities and parameters are employed in order to obtain Equations (1)–(7):

(8) $$\begin{array}{l} y=\frac{\bar{y}}{\varepsilon L},\;x=\frac{\bar{x}}{L},\;u=\frac{\bar{u}}{U},\;v=\frac{\bar{v}}{\varepsilon U},\;\varepsilon =\frac{H}{L},\;T=\frac{E(\bar{T}-T_{a})}{RT_{a}^{2}},\;P=\frac{\varepsilon^{2}L\bar{P}}{\mu_{f}U},\\ \;\beta =\frac{RT_{a}}{E},\;Ec=\frac{EU^{2}}{C_{pf}RT_{a}^{2}},\;Pe=\frac{\rho_{f}c_{pf}LU}{k_{f}},\;Re=\frac{UL}{\upsilon_{f}},\upsilon_{f}=\frac{\mu_{f}}{\rho_{f}},\;Bi=\frac{h_{f}H}{k_{f}},\\ Pr=\frac{\mu_{f}C_{pf}}{k},\;A_{1}=\frac{\rho_{nf}}{\rho_{f}},\;A_{2}=\frac{\mu_{nf}}{\mu_{f}},\;A_{3}=\frac{{\left(\rho C_{p}\right)}_{nf}}{{\left(\rho C_{p}\right)}_{f}},\;A_{4}=\frac{k_{nf}}{k_{f}},\;\lambda =\frac{QC_{0}BEH^{2}}{RT_{a}^{2}k_{f}}, \end{array}$$

where (*u*, *v*) are the velocity components of the nanolubricant in the (*x*, *y*) directions, respectively, *h_f_* is the heat coefficient, *U* is the mean velocity, *T* is the temperature, *ρ_nf_* is the nanolubricant density, *μ_nf_* is the nanolubricant dynamic viscosity, *k_nf_* is the nanolubricant thermal conductivity, *(ρC_p_)_nf_* is the nanolubricant heat capacitance, *Q* the heat of reaction, *B* the rate constant, *E* the activation energy, *R* the universal gas constant, *C*_0_ the initial concentration of the reactant species $\bar{P}$ is the pressure, *Pe* is the Peclet number, *Ec* is the Eckert number, *Pr* is the Prandtl number, *Bi* is the Biot number, *β* variable viscosity activation energy parameter, *λ* is the Frank–Kamenetskii parameter and *Re* is the Reynolds number. Since the microchannel is narrow with a very small aspect ratio 0 < *ε* << 1, for low Reynolds number flow, the lubrication approximation based on an asymptotic simplification of the governing dimensionless equations is invoked. From this vantage point, Equations (2)–(5) may be reduced to

(9) $$0=-\frac{\partial p}{\partial x}+A_{2}\frac{\partial }{\partial y}\left(e^{\frac{1}{\beta (1+\beta T)}}\frac{\partial u}{\partial y}\right)+O\left(\varepsilon^{2}\right),$$

(10) $$0=\frac{\partial p}{\partial y}+O\left(\varepsilon^{2}\right),$$

(11) $$0=A_{4}\frac{\partial^{2}T}{\partial y^{2}}+A_{2}Ec\mathrm{Pr}e^{\frac{1}{\beta (1+\beta T)}}{\left(\frac{\partial u}{\partial y}\right)}^{2}+\lambda e^{-\frac{1}{\beta (1+\beta T)}}+O(\varepsilon^{2}).$$

Following [5,10,18,20], the thermophysical expressions for variable viscosity reactive nanolubricant with respect to the nanoparticles are given as follows

(12) $$\;\rho_{nf}=(1-\phi )\rho_{f}+\phi \rho_{s},\mu_{nf}=\frac{\mu_{f}e^{\frac{1}{\beta (1+\beta T)}}}{{\left(1-\phi \right)}^{2.5}},\;\frac{k_{nf}}{k_{f}}=\frac{k_{s}+2k_{f}-2\phi (k_{f}-k_{s})}{k_{s}+2k_{f}+\phi (k_{f}-k_{s})},$$

where $\rho_{f}$ is the density of the PAO lubricant, $\rho_{s}$ is the density of the solid nanoparticle, *k_f_* is the PAO lubricant thermal conductivity, *k_s_* is the nanoparticle thermal conductivity, *ϕ* is the TiO_2_ nanoparticle volume fraction and *μ_f_* is the PAO lubricant dynamic viscosity. The physical properties of PAO lubricant together with TiO_2_ nanoparticles are listed in Table 1 below.

Simplifying Equations (9)–(11), we obtain

(13) $$\frac{d^{2}u}{dy^{2}}-\frac{1}{{\left(1+\beta T\right)}^{2}}\frac{du}{dy}\frac{dT}{dy}+\frac{G}{A_{2}}e^{-\frac{1}{\beta (1+\beta T)}}=0,$$

(14) $$\frac{d^{2}T}{dy^{2}}+\frac{A_{2}}{A_{4}}Ec\mathrm{Pr}{\left(\frac{du}{dy}\right)}^{2}e^{\frac{1}{\beta (1+\beta T)}}+\frac{\lambda }{A_{4}}e^{-\frac{1}{\beta (1+\beta T)}}=0,$$

where G=−dP/dx is the constant axial pressure gradient parameter. It is important to note that as *β*→∞, the model Equations (13) and (14) reduced to that of a constant viscosity TiO_2_-PAO nanolubricant flow in a microchannel with constant heat source whose exact solution based on the boundary conditions in Equations (6) and (7) can be easily obtained as

(15) $$u(y)=\frac{G}{2A_{2}}(y-y^{2}),$$

(16) $$T(y)=\frac{Ec\mathrm{Pr}G^{2}}{192A_{2}A_{4}}\left[1-{\left(2y-1\right)}^{4}\right]-\frac{\lambda }{2A_{4}}y^{2}+\frac{\left(A_{4}Ec\mathrm{Pr}G^{2}+24A_{2}A_{4}\lambda +12A_{2}Bi\lambda \right)}{24A_{2}A_{4}(A_{4}+Bi)}y.$$

In the following sections, Equations (13) and (14) together with the associated boundary conditions in Equations (6) and (7) are solved numerically via the shooting method with Runge–Kutta–Fehlberg integration scheme. Other quantities of engineering interest are the skin friction coefficients (*C_f_*) and Nusselt number (*Nu*) which are given as

(17) ReεCf=A2e1β(1+βT)dudy|y=0,1, Nu=−A4dTdy|y=0,1,

where $C_{f}=\frac{\tau_{w}}{\rho_{f}U^{2}},Nu=\frac{EHq_{w}}{k_{f}RT_{a}^{2}},\tau_{w}=\mu_{nf}\frac{\partial \bar{u}}{\partial \bar{y}},q_{w}=-k_{nf}\frac{\partial \bar{T}}{\partial \bar{y}}.$

## 3. Numerical Procedure

The dimensionless Equations (13) and (14) coupled with the boundary conditions in Equations (6) and (7) are nonlinear boundary value problems (BVP). We transformed these equations into a set of nonlinear first-order ordinary differential equations with some unknown initial conditions to be calculated by shooting technique [22]. Let

(18) $$u=x_{1},\;u^{\prime }=x_{2},\;T=x_{3},\;T^{\prime }=x_{4}.$$

The governing equations then become

(19) $$\begin{array}{c} {x^{\prime }}_{1}=x_{2},\;{x^{\prime }}_{2}=\frac{x_{2}x_{4}}{{\left(1+\beta x_{3}\right)}^{2}}-\frac{G}{A_{2}}e^{-\frac{1}{\beta (1+\beta x_{3})}},\\ {x^{\prime }}_{3}=x_{4},\;{x^{\prime }}_{4}=-\frac{A_{2}}{A_{4}}Ec\mathrm{Pr}x_{2}^{2}e^{\frac{1}{\beta (1+\beta x_{3})}}-\frac{\lambda }{A_{4}}e^{-\frac{1}{\beta (1+\beta x_{3})}} \end{array}\},$$

with the corresponding initial conditions as

(20) $$x_{1}(0)=0,\;x_{2}(0)=a_{1},\;x_{3}(0)=0,\;x_{4}(0)=a_{2}.$$

The values for *a*_1_ and *a*_2_ in Equation (20) are first guessed and then determined accurately with the shooting method via Newton–Raphson’s technique for each set of parameter values in Equation (19). Thereafter, Runge–Kutta–Fehlberg integration scheme [22] is then employed to tackle the resulting initial value problem numerically with step size ∆η = 0.01. From the numerical solution for velocity and temperature profiles, we compute the values for the skin friction (*C_f_*) and the Nusselt number (*Nu*) as given by Equation (17).

## 4. Results and Discussion

In this section, the effects of various emerging thermophysical parameters on the reactive TiO_2_-PAO variable viscosity nanolubricant velocity, temperature, skin friction, and Nusselt number in the microchannel are quantitatively discussed. The Prandtl number of PAO lubricants typically falls within the range of 10–15 depending on its composition, viscosity, and other factors [1]. In this study Pr = 12 is taken for PAO lubricant in the computational results. In order to validate the accuracy of our numerical procedure, the numerical results obtained for velocity and temperature profiles when *β*→∞ (i.e., a constant viscosity TiO_2_-PAO nanolubricant with constant heat source) are compared with their corresponding exact solutions displayed in Equations (15) and (16). A very excellent agreement is achieved as depicted in Table 2. This undoubtedly attests to the accuracy of our numerical procedure and the obtained results. 

### 4.1. Effects of Parameter Variation on Velocity Profiles

**Figure 2:**
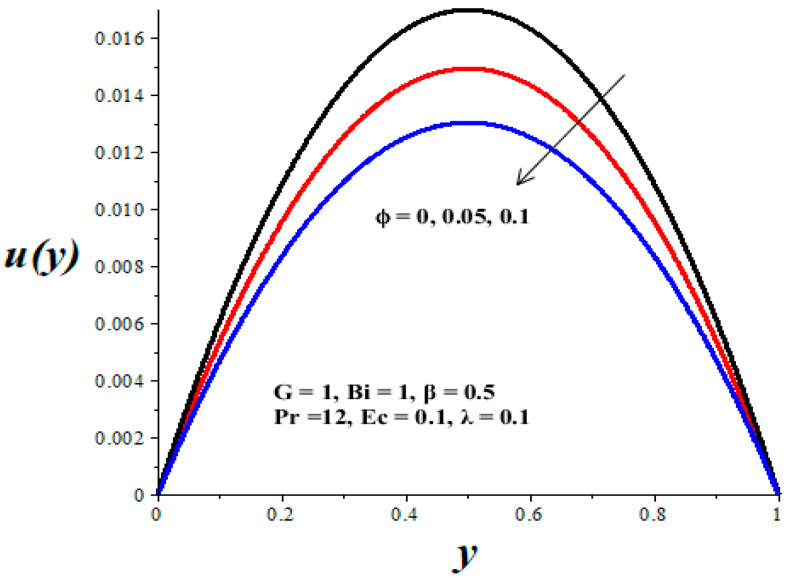
Effects of *ϕ* on *u*(*y*), black (*ϕ* = 0), red (*ϕ* = 0.05), blue (*ϕ* = 0.1).

**Figure 3:**
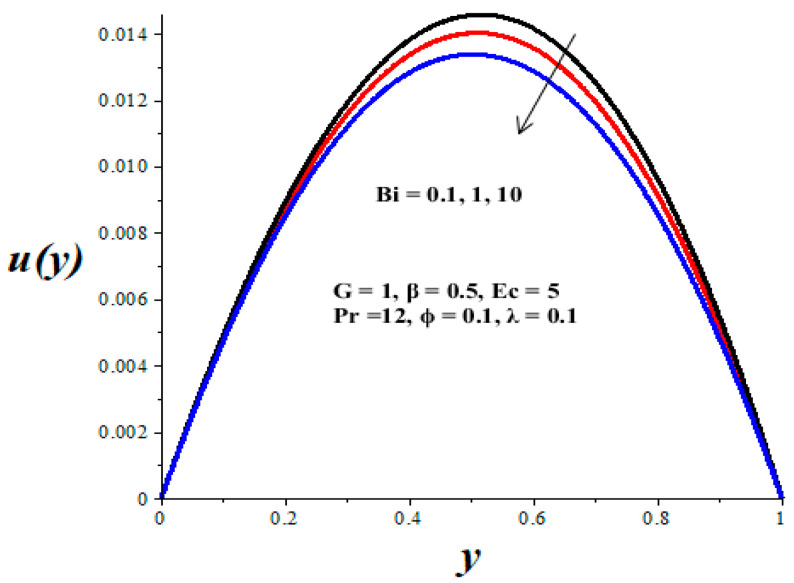
Effects of *Bi* on *u*(*y*), black (*Bi* = 0.1), red (*Bi* = 1), blue (*Bi* = 10).

**Figure 4:**
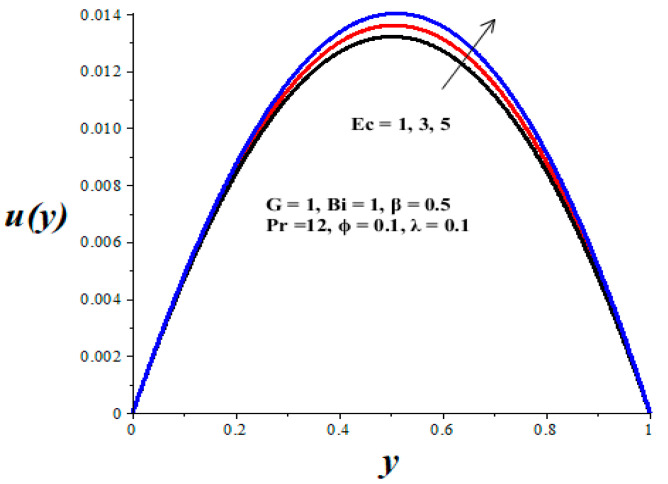
Effects of *Ec* on *u*(*y*), black (*Ec* = 1), red (*Ec* = 3), blue (*Ec* = 5).

**Figure 5:**
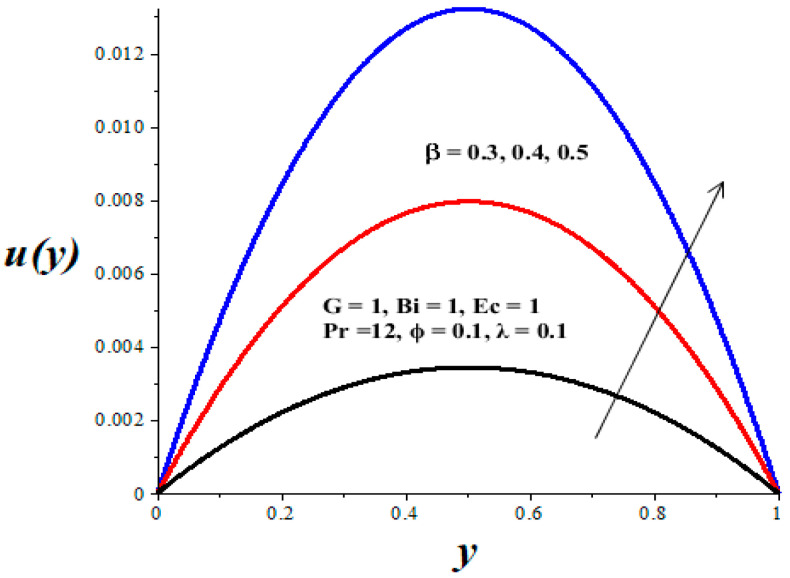
Effects of *β* on *u*(*y*), black (*β* = 0.3), red (*β* = 0.4), blue (*β* = 0.5).

**Figure 6:**
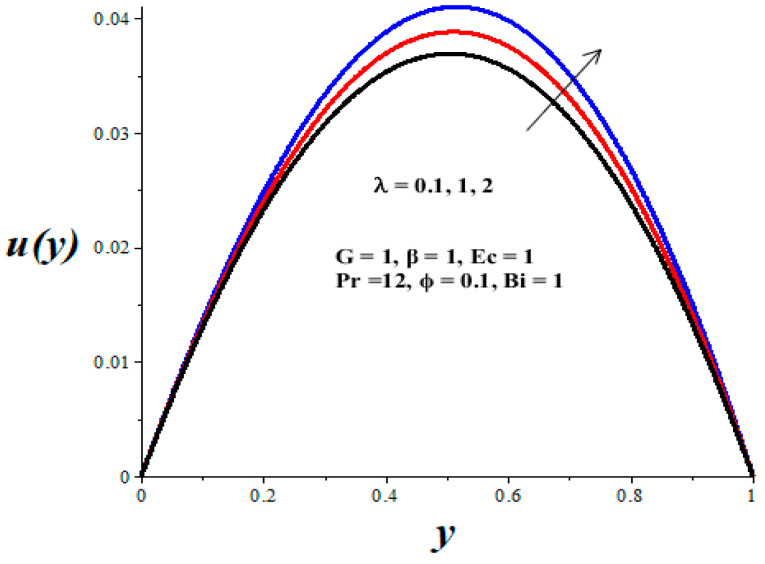
Effects of *λ* on *u*(*y*), black (*λ* = 0.1), red (*λ* = 1), blue (*λ* = 2).

Figure 2, Figure 3, Figure 4, Figure 5 and Figure 6 show the parameter effects on the variable viscosity reactive TiO_2_-PAO nanolubricant velocity profiles. In all the figures, the profile is Poiseuille parabolic with zero values at the walls and its peak value within the core region of the microchannel. Figure 2 and Figure 3 reveal a drop in the nanolubricant velocity with a rising value of TiO_2_ nanoparticles volume fraction and Biot number. This can be ascribed to a slight elevation in the viscosity of nanolubricant due to the presence of nanoparticles and the convective heat loss; consequently, the nanolubricant velocity lessens. In Figure 4, Figure 5 and Figure 6, it is interesting to note that the nanolubricant velocity profiles are enhanced with a rise in the values of the Eckert number (*Ec*), variable viscosity activation energy parameter (*β*), and the Frank–Kamenetskii parameter (*λ*). As the values of these parameters increase, the nanolubricant temperature rises and its viscosity drops, leading to an upsurge in the flow rate.

### 4.2. Effects of Parameters Variation on Temperature Profiles

**Figure 7:**
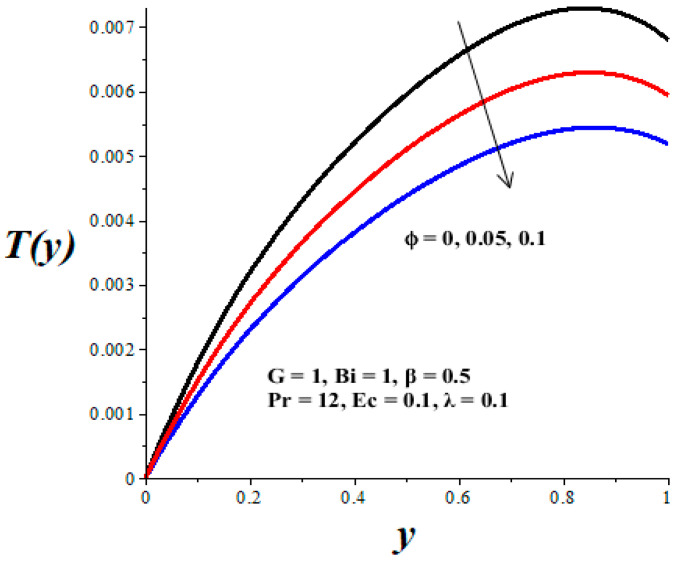
Effects of *ϕ* on *T*(*y*), black (*ϕ* = 0), red (*ϕ* = 0.05), blue (*ϕ* = 0.1).

**Figure 8:**
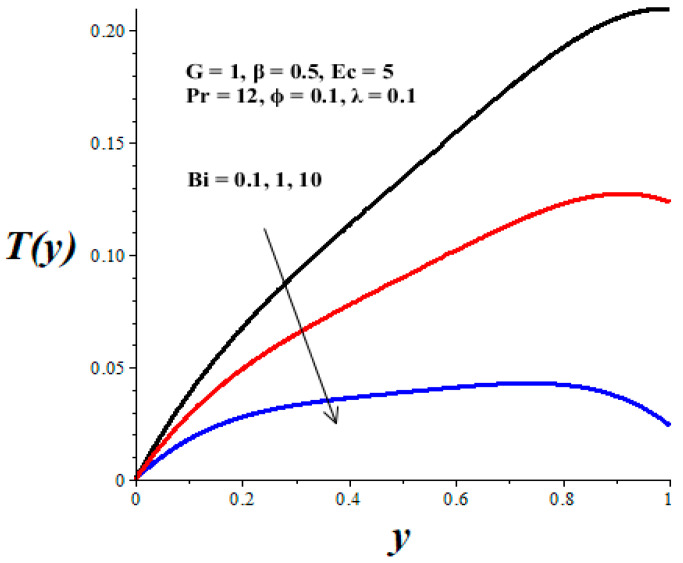
Effects of *Bi* on *T*(*y*), black (*Bi* = 0.1), red (*Bi* = 1), blue (*Bi* = 10).

**Figure 9:**
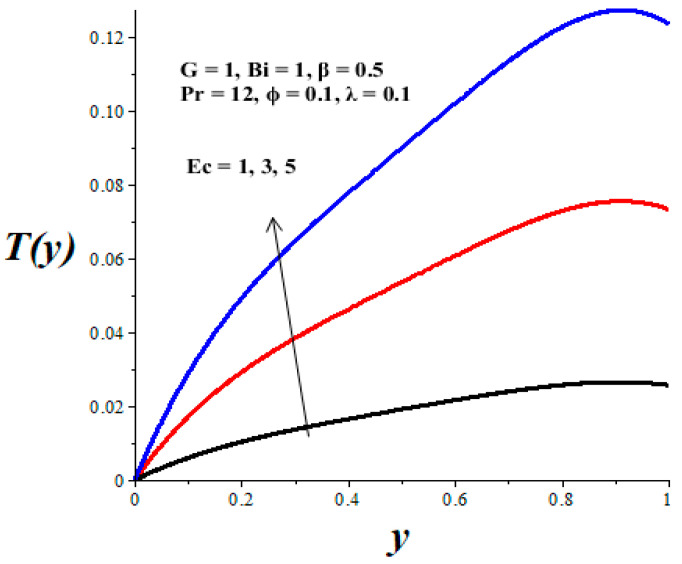
Effects of *Ec* on *T*(*y*), black (*Ec* = 1), red (*Ec* = 3), blue (*Ec* = 5).

**Figure 10:**
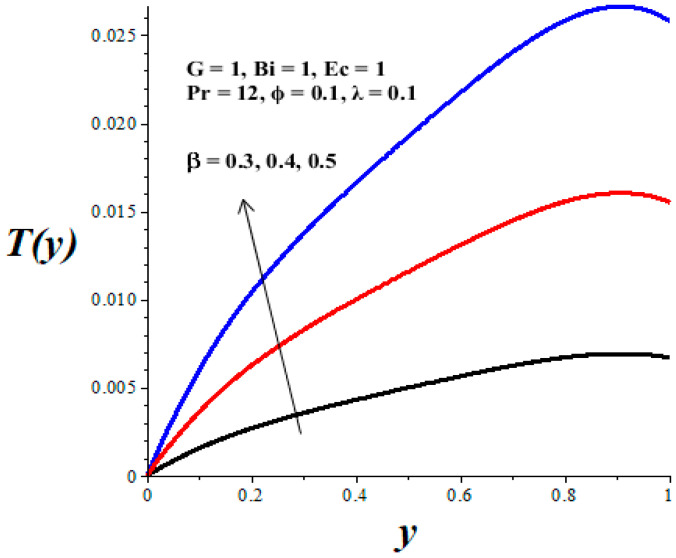
Effects of *β* on *T*(*y*), black (*β* = 0.3), red (*β* = 0.4), blue (*β* = 0.5).

**Figure 11:**
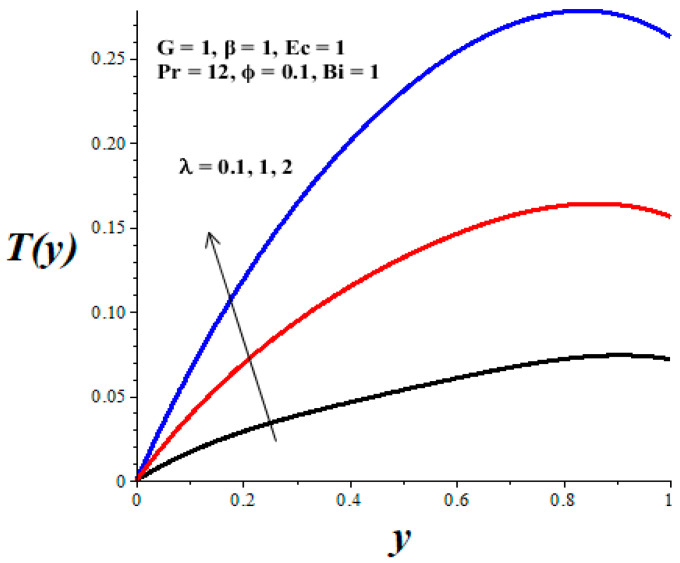
Effects of *λ* on *T*(*y*), black (*λ* = 0.1), red (*λ* = 1), blue (*λ* = 2).

The impacts of emerging parameters on the reactive nanolubricant temperature are displayed in Figure 7, Figure 8, Figure 9, Figure 10 and Figure 11. It is noteworthy that the nanolubricant temperature gradually increases from the lower wall, attained its pick value within the microchannel, and drops slightly at the upper wall due to convective heat loss to the ambient surrounding. Figure 7 and Figure 8 reflect a drop in the nanolubricant temperature with enhancing values of nanoparticles volume fraction and Biot number. This can be attributed to a rise in transfer rate and heat loss to the ambient surroundings; consequently, the nanolubricant temperature decreases. The behaviour of temperature profiles due to enhancing values of the Frank–Kamenetskii parameter (*λ*), Eckert number (*Ec*), and variable viscosity activation energy parameter (*β*) are presented in Figure 9, Figure 10 and Figure 11. The growing values of these parameters augment the nanolubricant temperature. This is expected, since a rise in exothermic Arrhenius kinetics and viscous dissipation due to a decrease in viscosity boosts the internal heat generation within the nanolubricant, leading to an elevation in temperature profiles.

### 4.3. Effects of Parameters Variation on Skin Friction

**Figure 12:**
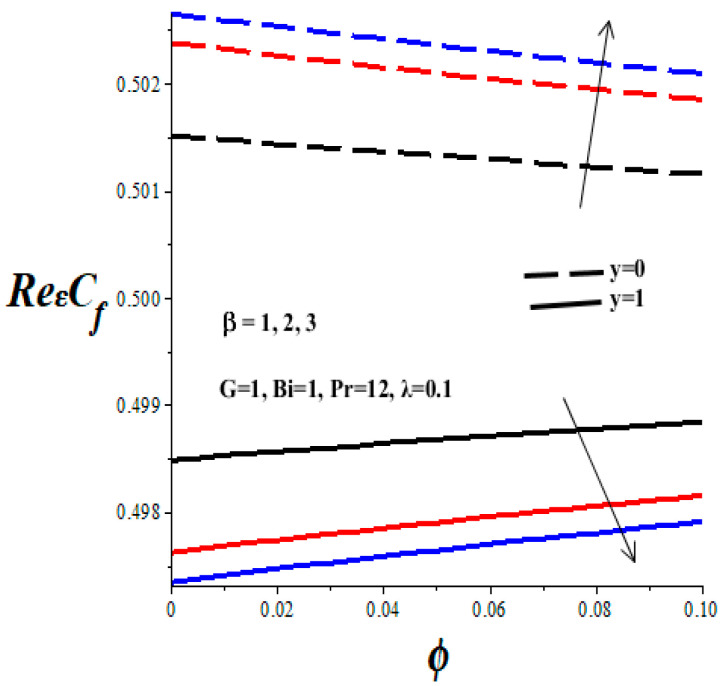
Effects of *ϕ* and *β* on skin friction, black (*β* = 1), red (*β* = 2), blue (*β* = 3), line (*y* = 1), dash (*y* = 0).

**Figure 13:**
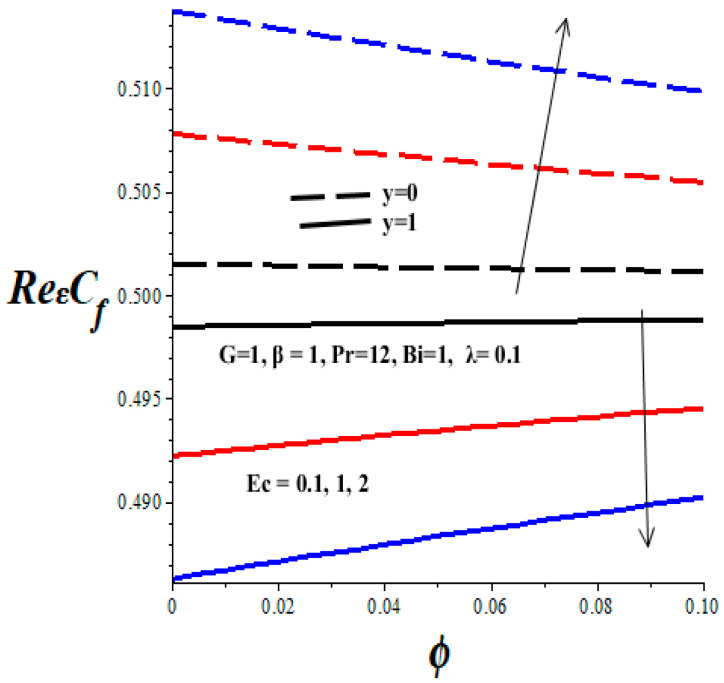
Effects of *Ec* on skin friction, black (*Ec* = 0.1), red (*Ec* = 1), blue (*Ec* = 2), line (*y* = 1), dash (*y* = 0).

**Figure 14:**
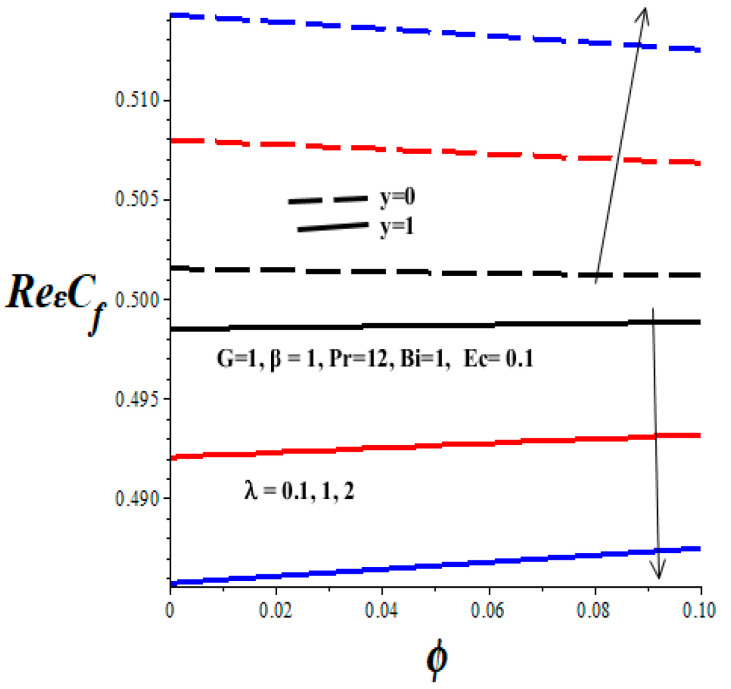
Effects of *λ* on skin friction, black (*λ* = 0.1), red (*λ* = 1), blue (*λ* = 2), line (*y* = 1), dash (*y* = 0).

**Figure 15:**
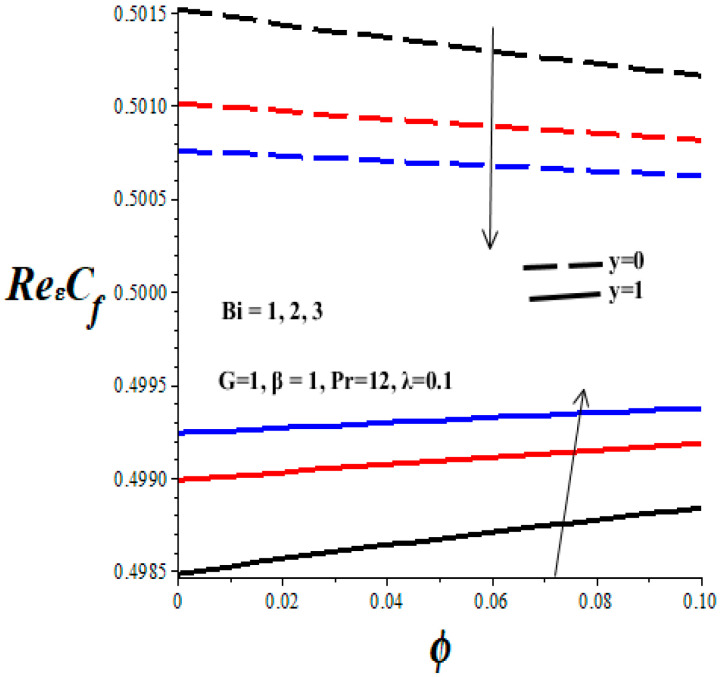
Effects of *Bi* on skin friction, black (*Bi* = 1), red (*Bi* = 2), blue (*Bi* = 3)*,* line (*y* = 1), dash (*y* = 0).

Figure 12, Figure 13, Figure 14 and Figure 15 display the impacts of emerging thermophysical parameters on the skin friction both at the lower and upper walls of the microchannel. Generally, the skin friction drops at the lower wall but rises at the upper wall with an elevation in TiO_2_ nanoparticles additive in the PAO nanolubricant. Moreover, an increase in the values of the Frank–Kamenetskii parameter (*λ*), Eckert number (*Ec*), and variable viscosity activation energy parameter (*β*) augments the skin friction at the lower wall and lessens the skin friction at the upper wall (see Figure 12, Figure 13 and Figure 14). The value of skin friction diminishes at the lower wall but rises at the upper wall with escalating values of Biot number (*Bi*) due to convective heat loss to the ambient surrounding, as depicted in Figure 15.

### 4.4. Nusselt Number and Thermal Criticality

Table 3 shows the parameter variation effects on the thermal critical Frank–Kamenetskii parameter *λ_c_*. The concept of thermal criticality is extremely important from an application point of view. This characterises the thermal stability criticality conditions in the flow field for a reactive TiO_2_-PAO nanolubricant under consideration and the onset of the thermal runaway phenomenon. It is noteworthy that the magnitude of *λ_c_* increases with an upsurge in parameters *ϕ* and *Bi*, but lessens with a rise in *β* and *Ec* parameter values. Undoubtedly, an increase in the value of *λ_c_* improves the nanolubricant thermal stability while a decrease in *λ_c_* heightens the onset of thermal runaway in the flow field and enhances the development of nanolubricant ineffectiveness. This evidently confirms that the TiO_2_ nanoparticles additive for PAO lubricant improves its thermal stability and enhances engine efficiency during operation. 

**Figure 16:**
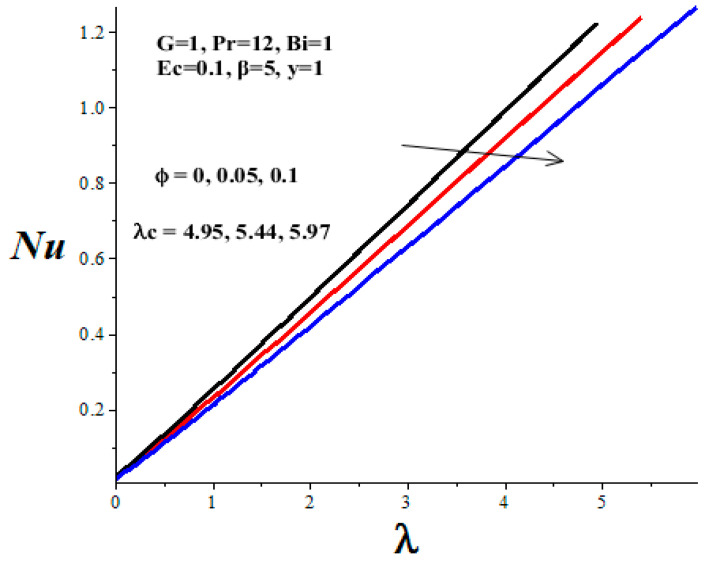
Effects of *ϕ* and *λ* on Nusselt number, black (*ϕ* = 0), red (*ϕ* = 0.05), blue (*ϕ* = 0.1).

**Figure 17:**
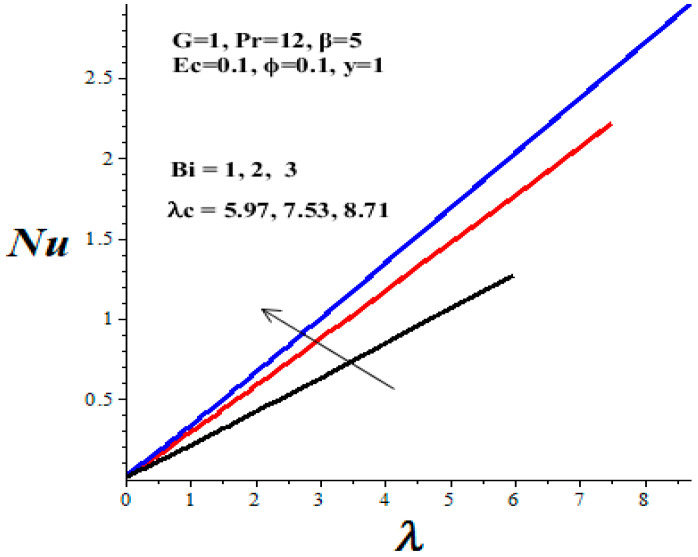
Effects of *Bi* on Nusselt number, black (*Bi* = 1), red (*Bi* = 2), blue (*Bi* = 3).

**Figure 18:**
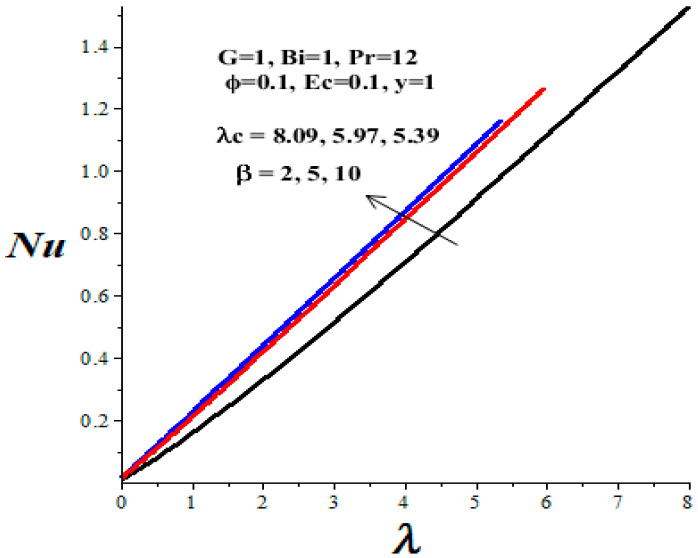
Effects of *β* on Nusselt number, black (*β* = 2), red (*β* = 5), blue (*β* = 10).

**Figure 19:**
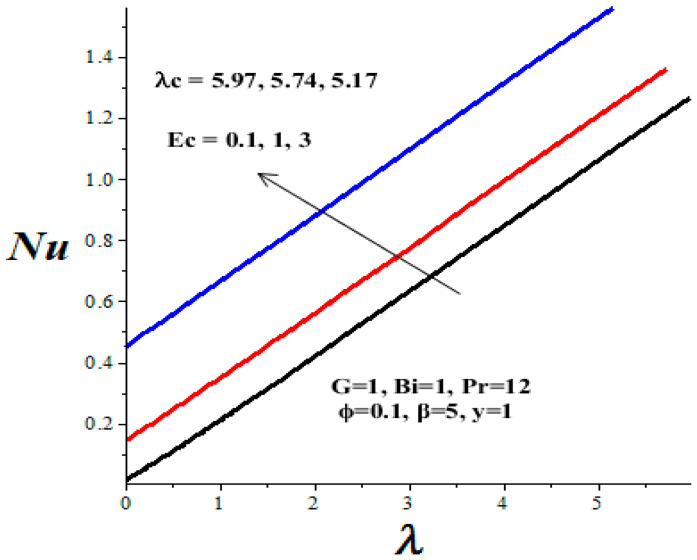
Effects of *Ec* on Nusselt number, black (*Ec* = 0.1), red (*Ec* = 1), blue (*Ec* = 3).

Figure 16, Figure 17, Figure 18 and Figure 19 depict the effects of thermophysical parameters on the Nusselt number at the upper wall of the microchannel. A critical value *λ_c_* exists such that, for 0 ≤ *λ* < *λ_c_*, the reactive TiO_2_-PAO nanolubricant is thermally stable (see Table 3). When *λ_c_* < *λ*, the system has no real solution and displays a classical form, indicating thermal runaway. It is found that the increasing values of the Frank–Kamenetskii parameter (*λ*), nanoparticles volume fraction (*ϕ*), and Biot number (*Bi*) tend to boost the value of the Nusselt number. The value of the Nusselt number diminishes with escalating values of the Eckert number (*Ec*) and variable viscosity activation energy parameter (*β*). Moreover, a drop in the nanolubricant viscosity with temperature coupled with a rise in viscous dissipation enhances its activation energy. Consequently, both the Nusselt number and the thermal critical values are lessened, as shown in Figure 18 and Figure 19 and Table 3. This heightened the nanolubricant vulnerability to thermal runaway during the flow process due to excessive heat accumulation. Meanwhile, a rise in TiO_2_ nanoparticles additive in PAO nanolubricant coupled with exothermic Arrhenius kinetics and convective heat loss to the ambient surrounding boosts the Nusselt number and the thermal critical values, as clearly shown in Figure 16 and Figure 17 and Table 3.

## 5. Concluding Remarks

A nonlinear mathematical model for Poiseuille flow of a reactive variable viscosity TiO_2_-PAO nanolubricant in a microchannel with heat transfer enhancement characteristics is developed and numerically tackled via the shooting method with Runge–Kutta–Fehlberg integration scheme. Pertinent results showing the effects of emerging thermophysical parameters on the velocity, temperature, skin friction, Nusselt number and thermal stability criteria were obtained. Our dimensionless results depict the global effects of exothermic kinetic and other parameters on nanolubrication in microchannels. The key findings of this work are as follows:

- The velocity profiles are enhanced with *Ec*, *β*, and *λ* but lessened with *ϕ* and *Bi*;
- The growing values of *Ec*, *β*, and *λ* augment the temperature profiles while a rise in *ϕ* and *Bi* lessen it;
- A rise in *Ec*, *β*, *λ*, and *ϕ* lessened the skin friction at the upper wall but heightened it at the lower wall. As Bi increases, the skin friction drops at the lower wall but intensifies at the upper wall;
- The value of the Nusselt number diminished with rising values of *Ec* and *β* but enhanced with the growing values of *ϕ*, *λ*, and *Bi*;
- A thermal critical value *λ_c_* exists such that, for 0 ≤ *λ < λ_c_* the reactive TiO_2_-PAO nanolubricant is thermally stable. When *λ_c_ < λ* the system has no real solution and displays a classical form indicating thermal runaway.

## Figures and Tables

**Table 1:** Nanoparticles and base fluid thermophysical properties [5,10,18].

Physical Properties	*ρ* (kg/m^3^)	*C_p_* (J/kg·K)	*k* (W/m·K)
PAO	798	2303	0.143
TiO_2_	4010	690	8.7

**Table 2:** Comparison between the exact and numerical results when *β*→∞, *Bi* = 1, *G* = 1, *Pr* = 12, *Ec* = 1, *λ* = 1, *ϕ* = 0.1.

y	u(y) Exact	u(y) Numerical	T(y) Exact	T(y) Numerical
0	0.00000000	0.0000000	0.0000000	0.0000000
0.1	0.03457950	0.03457951	0.0939346	0.0939347
0.2	0.06147467	0.06147468	0.1689389	0.1689390
0.3	0.08068551	0.08068551	0.2299185	0.2299186
0.4	0.09221201	0.09221202	0.2803778	0.2803778
0.5	0.09605418	0.09605418	0.3224191	0.3224192
0.6	0.09221201	0.09221202	0.3567434	0.3567435
0.7	0.08068551	0.08068551	0.3826498	0.3826499
0.8	0.06147467	0.06147468	0.3980358	0.3980358
0.9	0.03457950	0.03457951	0.3993972	0.3993973
1.0	0.00000000	0.00000000	0.3818282	0.3818282

**Table 3:** Computations showing the effect of parameter variation on thermal stability critical Frank–Kamenetskii parameter for PAO-TiO_2_ reactive lubricant (G = 1, Pr = 12).

*ϕ*	*β*	*Ec*	*Bi*	*λ_c_*
0	5	0.1	1	4.95294
0.05	5	0.1	1	5.44322
0.1	5	0.1	1	5.97406
0.1	10	0.1	1	5.39884
0.1	2	0.1	1	8.09072
0.1	5	1	1	5.74703
0.1	5	3	1	5.17685
0.1	5	0.1	2	7.53357
0.1	5	0.1	3	8.71568

## Data Availability

All data generated or analyzed are included in the article.
